# Novel Methods of Incorporating Time in Longitudinal Multivariate Analysis Reveals Hidden Associations With Disease Activity in Systemic Lupus Erythematosus

**DOI:** 10.3389/fimmu.2019.01649

**Published:** 2019-07-17

**Authors:** Hieu T. Nim, Kathryn Connelly, Fabien B. Vincent, François Petitjean, Alberta Hoi, Rachel Koelmeyer, Sarah E. Boyd, Eric F. Morand

**Affiliations:** ^1^Data Science & AI, Faculty of Information Technology, Monash University, Clayton, VIC, Australia; ^2^Centre for Inflammatory Diseases, School of Clinical Sciences at Monash Health, Monash University, Melbourne, VIC, Australia

**Keywords:** systemic lupus erythematosus, biomarkers, clustering, longitudinal analysis, regression models

## Abstract

**Objective:** Systemic lupus erythematosus (SLE) is a multisystem autoimmune disease. SLE is characterized by high inter-patient variability, including fluctuations over time, a factor which most biomarker studies omit from consideration. We investigated relationships between disease activity and biomarker expression in SLE, using novel methods to control for time-dependent variability, in a proof-of-concept study to evaluate whether doing so revealed additional information.

**Methods:** We measured 4 serum biomarkers (MIF, CCL2, CCL19, and CXCL10) and 13 routine clinical laboratory parameters, alongside disease activity measured by the SLE disease activity index-2k (SLEDAI-2k), collected longitudinally. We analyzed these data with unsupervised learning methods via ensemble clustering, incorporating temporal relationships using dynamic time warping for distance metric calculation.

**Results:** Data from 843 visits in 110 patients (median age 47, 83% female) demonstrated highly heterogeneous time-dependent relationships between disease activity and biomarkers. Unbiased magnitude-based hierarchical clustering of biomarker expression levels isolated a patient subset (*n* = 9) with distinctively heterogeneous expression of the 17 biological parameters, and who had MIF, CCL2, CCL19, and CXCL10 levels that were higher and more strongly associated with disease activity, based on leave-one-out cross-validated regression analysis. In the remaining subgroup, a time-dependent regression model revealed significantly stronger predictive power of biomarkers for disease activity, compared to a time-agnostic regression model. Despite no significant difference in simple magnitude, using dynamic time warping analysis to align longitudinal profiles revealed a large subset (*n* = 69) with significantly stronger associations between biological parameters and disease activity. This subgroup had significantly lower flare rates, disease activity and damage scores, suggesting this clustering is clinically meaningful.

**Conclusions:** These results suggest associations between biological parameters and disease activity in SLE exist in a multi-dimensional time-dependent pattern, with implications for the analysis of biomarkers in SLE often used to identify therapeutic targets. Novel methods to analyse high-dimensional data and control for time-dependent variability may have broad utility in the study complex relationships between clinical and biological parameters.

## Introduction

Systemic lupus erythematosus (SLE) is an archetypal multisystem autoimmune disease, which results in a marked loss of life expectancy, a fact that has changed little in recent decades as “breakthrough” treatments have not emerged (1, 2). Although patients diagnosed with SLE share autoimmunity to nucleic acids and immune-mediated tissue damage, SLE is characterized by high inter-patient variability in terms of clinical and biological characteristics, suggesting value in identifying biologically-defined subsets of patients for the application of targeted therapies (3). Studies of biomarkers such as serum cytokines have been used to in these attempts, but to date robust relationships between such analytes and disease activity measures have been elusive (4, 5).

New ways to identify biological subsets of SLE patients are urgently needed, given the repeated failure of targeted therapies in clinical trials of biologically unstratified patients (6). A notable characteristic of SLE is its volatile course over time, with unpredictable clinical relapse and remission cycles. The marked time-dependent volatility of SLE, and the likely existence of distinct subsets within the disease, have typically not been addressed in biomarker studies; associations that have been identified between biomarkers and disease activity are modest at best, suggesting that dynamic relationships between biological data and disease activity may be missed by traditional analytical approaches and clouded by the pooling of heterogeneous patients. This is especially true of cross-sectional data, and multiple studies have underscored the need to study associations in longitudinal fashion (7, 8), thus allowing consideration of the temporal or time dimension.

In this proof-of-concept study, we test the hypothesis that novel methods of data analysis using unsupervised learning may reveal patient subsets, and associations with disease activity, that are hidden when using traditional data analysis. Previously, we have applied more familiar longitudinal analysis methods in our well-characterized lupus cohort with general estimating equations (9). More sophisticated analysis techniques such as unsupervised learning methods have shown great promise in various biomedical data sets (10, 11), including more recently as a novel means of analyzing complex data in SLE (7, 12, 13). These machine learning methods draw inferences from unclassified datasets to identify latent patterns; for example, cluster analyses are increasingly used for exploratory data analysis to identify groupings that may be hidden when using standard methods. Recently, Toro-Dominguez et al. (13), using unsupervised learning methods, demonstrated that molecular clusters could be identified by the longitudinal correlations between blood transcriptome data and disease activity in SLE, which in turn were associated with neutrophil and lymphocyte numbers and the development of clinical features such as proliferative lupus nephritis.

Similarly, in this study we investigate, as a proof of concept study, whether the use of unsupervised learning, as a technique to overcome the challenges of the high-dimensional nature of clinical registry data, reveals data hidden when using traditional approaches. We focussed first on the simple magnitude of various biological parameters to identify patient clusters, and then on the temporal dimension, to determine the impact of time-dependent analysis on relationships between biomarkers and disease phenotype in SLE. The biological parameters included in our analysis are blood and urine markers routinely measured in SLE management, as well as four candidate immune biomarkers readily detected in patient samples (9), namely three type I interferon (IFN) inducible chemokines CCL19, CCL2, and CXCL10, which reflect IFN activity in SLE (14), and macrophage migration inhibitory factor (MIF), which has also been identified as a target for SLE (9). The goal of this study was therefore not to identify novel biomarkers, but to study whether the dimension of time adds value when analyzing biomarker data in SLE.

## Materials and Methods

### Patient Characteristics

Data were obtained for the period May 2007 to December 2012 from the Australian Lupus Registry (15), where SLE patients over 18 years old fulfilling the 1982 American College of Rheumatology (ACR) revised criteria (16) have been recruited and longitudinal clinical data and serum samples archived, as previously described (4, 5, 17). Patients with full clinical data capture, and matched serum samples from at least 3 visits, were selected for this study, with the first such visit defined as baseline, day 0. All patients gave written informed consent. This study was approved by the Human Research Ethics Committee, Monash Health (Monash Health HREC Reference 15526L).

### Data Pre-processing

Amongst the data collected at each visit, the present study investigated all 13 routinely collected clinical laboratory parameters [C-reactive protein (CRP), complement components C3 and C4, hemoglobin (Hb), total white cell count (WCC), platelets, neutrophils, lymphocytes, erythrocyte sedimentation rate (ESR), anti-double-stranded DNA antibodies (dsDNA), and urine protein/creatinine ratio (UPCR), urine WCC, and urine red cell count]. Anti-dsDNA results from different assays were converted to fold above upper limit normal (ULN) using the ULN value for the relevant assay. Serum concentrations of three type I IFN inducible chemokines (CCL19, CCL2, and CXCL10) and MIF were measured by ELISA, in serum samples collected at the same time as the clinical laboratory data and stored at −80°C as previously described (9). Disease activity was assessed at each visit using the SLEDAI-2k, as previously described (9). Patients with missing data, and patients with fewer than three clinic visits, were excluded. The 17 laboratory parameters were *z*-normalized (mean = 0, *sd* = 1), such that each parameter was normalized across all the patient records.

### Statistical Analysis

R software version 3.3.2 was used to perform all statistical analyses, in which all packages mentioned were part of the base distribution of R unless otherwise stated. Only the 17 laboratory measurements and the time dimension were included in the initial analysis. SLEDAI-2k was included in subsequent statistical analyses. Hierarchical clustering (agglomerative) was performed using the *hclust* R package, and patient groups (or clusters) produced by cutting the dendrogram at a specified height. The *heatmap* of patient distances was produced using the *heatmap.2* R package. Two-dimensional classical (metric) and isotonic multidimensional scaling (MDS) were performed using the *cmdscale* R package.

Dynamic time warping (DTW) distance for hierarchical clustering analysis was calculated based on alignment between two patient time-series, allowing the matching of patients with similar disease progression albeit at different time intervals. Alignment permits open ends for each time series. For each pair of patients *x* and *y*, the DTW distance was performed in the R environment using the *dtw* package, which minimizes the squared Euclidean distance between two time series, defined as:

$$\begin{array}{l} \arg\underset{p}{\underset{︸}{\min}}\left(\underset{k}{\sum }{\left(x_{k,m}-y_{k,n}\right)}^{2}\right) \end{array}$$

In the above equation, for each alignment *P*, the time point *m* of patient *x* is aligned with time point *n* of patient *y*, and *k* denotes the *k*th biological parameter. In the *dtw* R package, the search for the optimal alignment *P* can be performed efficiently using dynamic programming technique.

For internal clustering evaluating, connectivity and Dunn index were calculated using the *clValid* R package. Connectivity is numeric value indicating the connectedness of the clustering results, defined as:

$$\begin{array}{l} c=\overset{1}{\underset{i=n}{\sum }}\underset{j}{\sum }x_{ij}, \end{array}$$

where x_*ij*_ = 0 if variable *i* in the same cluster as its *j*th nearest neighbor, and x_*ij*_ = 1/*j* otherwise. Dunn index is defined as the ratio between the minimal inter-cluster distance to maximal intra-cluster distance:

$$\begin{array}{l} D=\frac{\min_{1\leq i<j\leq n}d_{ij}}{\max_{1\leq k\leq n}I_{k}}, \end{array}$$

where D is the Dunn index, d_*ij*_ is the inter-cluster distance between the *i*th and *j*th clusters, and I_*k*_ is the intra-cluster distance of the *k*th cluster. The distance matrix is obtained from the clustering analysis, which can be either Euclidean distance or DTW distance.

Multiple linear regression was performed with leave-one-patient-out (LOPO) partitioning for validation. Regression was performed first with all patients (*n* = 110), then with every individual cluster of patients identified by hierarchical clustering. Bootstrapping was performed using the *boot* R package, with 1,000 iterations of 80% training data and 20% test data. Error score was based on mean square Euclidean distance between each data point and the corresponding predicted value from the multiple linear regression. Logistic regression was used to assess the association of group labels with SLE disease characteristics and adverse outcomes. Unless otherwise specified, missing data was excluded from the analysis. Most variables had a low level of missing data. An α = 0.05 was set as the threshold for statistical significance in this analysis. For rare occurrence binary variables (<5% of the total number of patients), exact logistic regression was used to account for the small sample size. All analysis source code in R is available upon request; however patient data is only available to ALRB members.

## Results

### Heterogeneity of Clinical and Biomarker Relationships Among SLE Patients

The final dataset contained data and samples from 843 time points for 110 patients, whose characteristics are summarized in Table 1. At baseline, median (interquartile range) age and disease duration were 47 (38–56) and 14.5 (10–21) years, respectively. Across the observation period, time-adjusted mean SLEDAI-2K (AMS) was 4 (2–5), SLICC-SDI (SLE damage index) was 1 (0–2) and 84% of patients were received prednisolone, 96% antimalarials, 77% immunosuppressants, and 6% biologics.

**Table 1:** SLE patient demographic, clinical, and biological characteristics at baseline.

**Parameter**	**Descriptive statistics number (% of total *n* = 110)**
**Sociodemographic characteristics**	
**Sex**	
Female	91 (83%)
Male	19 (17%)
**Ethnicity**	
Caucasian	53 (48%)
Asian	53 (48%)
Other/Missing	4 (4%)
**Disease characteristics**	
**Age at diagnosis (years)**	
<18 years	13 (12%)
≥18 to <45 years	77 (70%)
≥45 years	20 (18%)
**Disease duration (years)**	
<10 years	40 (36%)
≥10 years	70 (64%)
**Organ involvement at diagnosis (ACR criteria)**	
Arthritis	73 (66%)
Discoid rash	16 (15%)
Haematologic disorder	63 (57%)
Immunological disorders	94 (85%)
Malar rash	49 (45%)
Neurologic disorder	14 (13%)
Oral ulcers	40 (36%)
Photosensitivity	35 (32%)
Renal disorder	45 (41%)
Serositis	51 (46%)
**Immunological features**	
Anti-Nuclear Antibody	108 (98%)
Anti-dsDNA	83 (75%)
Anti-Sm	19 (17%)
Low complement component 3 (C3)	84 (76%)
Low complement component 4 (C4)	88 (80%)
**Treatment**	
Prednisolone	92 (84%)
Hydroxychloroquine	106 (96%)
Immunosuppressants	85 (77%)
Biologics	7 (6%)

**Figure 1:**
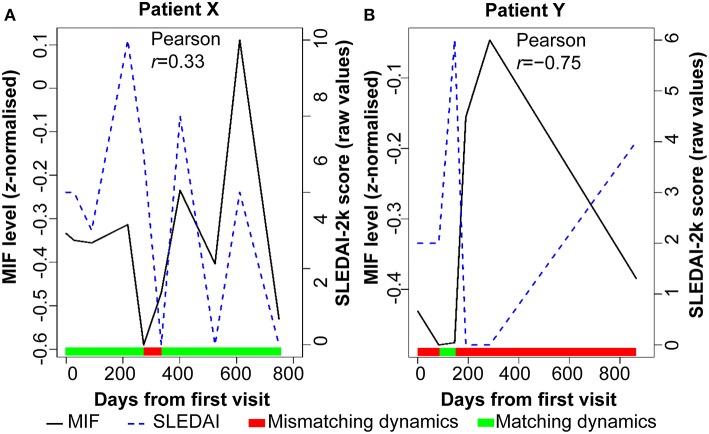
Disease activity and serum MIF levels for two SLE patients over time. The color bar indicates when the serum MIF and SLEDAI-2k dynamics are synchronized (green) or desynchronised (red). **(A)** Patient X has a moderate correlation between disease activity (SLEDAI-2k) and serum levels of MIF protein. **(B)** Patient Y has a complete lack of correlation between these two parameters. Serum MIF levels are shown as z-normalized values.

First, associations between biomarker and disease activity time courses of individual patients were examined one by one. Marked variation in these associations between individual patients was observed. Figure 1 shows the time course of serum MIF concentration and SLEDAI-2k, and matching dynamics of change between the two measures, for two individual patients as examples. Ninety-three percent similar dynamics were observed between serum MIF levels and SLEDAI-2k score in patient X, in whom MIF and SLEDAI-2k measured over time exhibit a positive correlation [Pearson correlation coefficient *r* = 0.33]. In contrast, patient Y showed no evidence of a relationship between MIF and SLEDAI-2k, with <10% similar dynamics between serum MIF levels and SLEDAI-2k. This exemplified the heterogeneity in biomarker-clinical state concordance over time among individual patients, suggesting that relationships may be hidden when examining these relationships in pooled patients and that distinct subsets of patients could be revealed if time-dependent associations were analyzed.

### Two Distinct SLE Patient Groups (Group 1 and Group 2) Are Defined by Magnitude-Based Clustering of Biological Parameters

**Figure 2:**
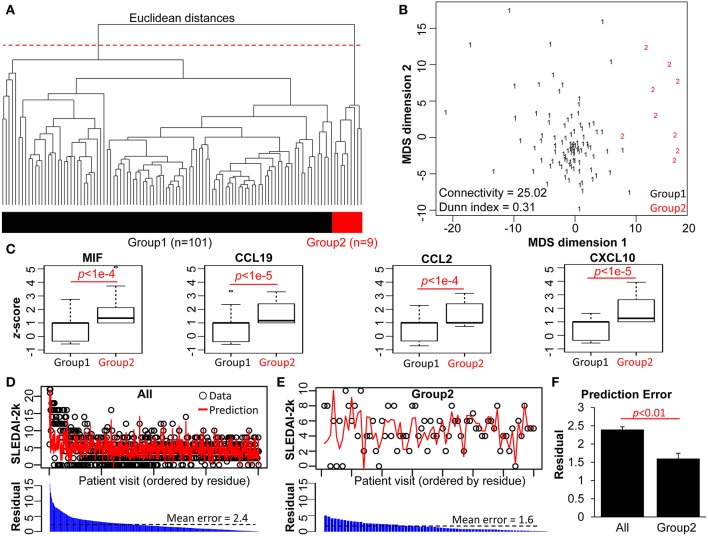
Magnitude-based clustering of SLE patients based on 17 biological parameters. **(A)** The dendrogram of the pairwise Euclidean distances of the 110 SLE patient pathology profiles. An arbitrary cut-off of 90% of the dendrogram height (red dashed line) produces two groups of patients (bi-colored bar). **(B)** Isotonic MDS plot of the Euclidean distances between the 110 patients. Group 2 (red) exhibits lower intra-cluster similarity compared to Group 1 (black). The Connectivity and Dunn indices (bottom left) indicate the quality of the clustering method. **(C)** Boxplot comparison of the two patient groups, based on z-normalized serum cytokine parameters. **(D,E)** Results from LOPO multiple linear regression to predict the disease activity (SLEDAI-2K) of each patient visit based on the blood and urinary parameters, performed on **(D)** all patients vs. **(E)** patients from Group 2. Black circles represent actual SLEDAI-2k values from the patient cohorts, while red lines represent the predictions from the LOPO linear regression model. All data points were arranged in descending order of the residuals. **(F)** Comparison of prediction error of Group 2 patients vs. all patients without grouping information. With group stratification, Group 2 exhibits strong power to predict SLEDAI-2K score, based on the low mean residual (absolute error between the predicted and actual SLEDAI-2K scores), compared to all data.

Next, we performed a clustering analysis to identify whether patient subsets could be grouped purely based on the multivariate relationships between the 17 measured biological parameters in each patient. To do this, we applied multivariate magnitude-based Euclidean distance to perform a pairwise comparison of the laboratory profiles and performed hierarchical clustering (agglomerative) on multivariate Euclidean distances. This allows identification of patient subgroups distinguished by shared features in the measured biological parameters. The distances and clusters from these analyses were visualized using a dendrogram and heat map (Figure 2A and Figure S1). Multiple clustering methods showed the likelihood of k = 2 groups in this dataset (Figure S2). Thus, cutting the dendrogram at 90% height level (red dashed line) delineates two groups of patients in the cohort. A classical (metric) multidimensional scaling (MDS) plot illustrates the distinct distribution of the groups (Figure 2B) in a visual manner: Group 1 (*n* = 101) is more aggregated, with a very high intragroup similarity, whereas Group 2 (*n* = 9) is more separated, with high intragroup variability. We also quantified the internal clustering quality using the metrics of connectivity (25.02, lower indicates better quality) and Dunn index (0.31, higher indicates better quality).

### SLE Patients in Group 2 Are Characterized by Significantly Higher Chemokine Levels

Clustering analyses as performed above occur in a multi-dimensional space, where the specific role of individual parameters in forming the identity of each group may not be readily apparent. We therefore assessed the individual parameters of the two patient groups. Complete data is provided in Table S1, and in Figure 2C and Figure S3 which visualize the distribution of the biological parameters with box-and-whisker plots. For the standard laboratory parameters, the differences between the two groups were not statistically significant (Figure S3). However, levels of serum MIF, CCL19, CCL2, and CXCL10 were each significantly higher in Group 2 compared to Group 1 (Figure 2C). This may explain the delineation of the two groups that was defined by hierarchical clustering. Clinical differences were also examined between the two groups, with patients in Group 2 having significantly more musculoskeletal, mucocutaneous and immunological disease features (Table S1).

### SLE Patients in Group 2 Are Characterized by a Strong Association of Disease Activity With the 17 Biological Parameters

To investigate the relationship between the 17 measured biological parameters and disease activity as assessed by the SLEDAI-2k, we performed multiple linear regression for the 110 patients at every time point (*n* = 843 data points), with LOPO cross-validation, to compare two scenarios: (1) patients were treated as a single group (All), and (2) patients were separated into the two groups defined by the hierarchical clustering (Figures 2D–F). Figures 2D,E show the actual (observed) SLEDAI-2k data point, and the corresponding predicted SLEDAI-2k value, as derived by fitting the multiple linear regression model with the remaining data points. The LOPO regression model showed no difference in SLEDAI-2k prediction in Group 1 when compared to all patients (Figure S4). This is partially due to the lack of intra-group diversity among Group 1 patients (Figure 2B), as well as the large size of this group (*n* = 101). In contrast, Group 2 exhibited significantly stronger power for the biological parameters to predict actual SLEDAI-2k, compared to the patient group as a whole (Figure 2F and Figures S7A–C). This suggests the ability for this approach to define distinct subsets of SLE patients with regard to clinical state-biomarker associations.

### Associations of Disease Activity With Temporal Changes in Biological Parameters—Incorporating the Time Dimension

**Figure 3:**
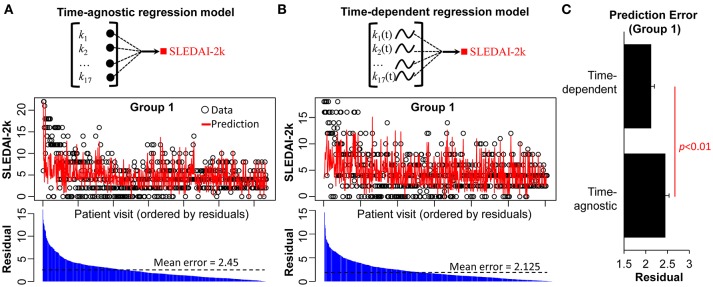
Predicted vs. actual SLEDAI-2K measures based on time-agnostic and time-dependent regression models. LOPO cross validation using **(A)** a time-agnostic regression model and **(B)** a time-dependent regression model. For each model, the predicted SLEDAI-2K of each patient visit is compared with the actual SLEDAI-2K. **(C)** Comparison between the prediction errors of time-agnostic vs. time-dependent regression model as applied to Group 1.

The regression analysis applied above was time-agnostic; the data points are aggregated into an unordered data matrix, meaning that patient visits are treated as independent of each other, and aggregated at the patient level. This traditional approach is incapable of determining time-dependent relationships between the variables in an individual patient, a significant limitation in a disease such as SLE. We therefore repeated our regression analysis in Group 1, but this time generated a time-dependent regression model. This was achieved by building the regression model linking the 17 biological parameters for each patient visit with the visit prior. The LOPO cross-validation in this time-dependent model (Figure 3A) showed a significant improvement in the association of the biological parameters with disease activity compared to the time-agnostic model (Figures 3B,C). The time-dependent regression model has higher degree of freedom (35 free parameters) compared to time-agnostic regression model (17 free parameters). While more complex models may suffer from overfitting and poor test results, this was not the case for Group 1. These results demonstrate how considering the time dimension in analysis may improve the ability to detect associations of biomarkers with disease activity.

### Dynamic Time Warping (DTW) Analysis of Group 1 Shows Significant Association Between the Time Course of Biological Parameters and Disease Activity

We next sought to identify whether patient-stratifying biomarker patterns exist that are concealed when time is not considered. We used Dynamic Time Warping (DTW) analysis, a method which operates non-linear distortions of the time-axis to realign the time-course of the readings (18), allowing for the measurement of similarity between two temporal sequences and controlling for variation in tempo which could otherwise obscure associations.

**Figure 4:**
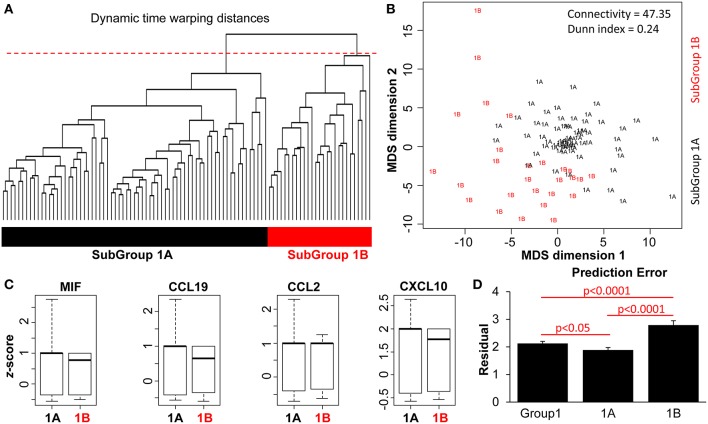
DTW clustering analysis as applied to patients in Group 1 (*n* = 101). **(A)** A dendrogram of the pairwise Euclidean distances of the patient pathology profiles in Group 1 (*n* = 101). An arbitrary cut-off of 90% height was applied to the dendrogram (red dashed line), to produce two patient subgroups (color bars). **(B)** Isotonic MDS plot of the Euclidean distances between the 101 patients, with Subgroup 1A (*n* = 69) and Subgroup 1B (*n* = 32), indicated by text and colors. Connectivity and Dunn index metrics (top right) indicate the quality of the clustering method. **(C)** Boxplot comparison between the two subgroups of patients based on z-normalized serum cytokine parameters. **(D)** Comparison of prediction error with and without subgrouping information. Bar plots represent results from LOPO multiple linear regression to predict the SLEDAI-2K of each patient visit based on the blood and urinary parameters, performed on all Group 1 patients vs. Subgroup 1A and 1B patients.

Firstly, we computed multivariate DTW distance to perform a pairwise comparison of the profiles of patients in Group 1 (*n* = 101). Then, to the time-warped data, we once again applied hierarchical clustering (agglomerative) to generate a dendrogram (Figure 4A). Multiple clustering methods showed the highest likelihood of *k* = 2 subgroups in this dataset (Figures S5). Thus, cutting the dendrogram at the same 90% (red dashed line) further delineated two patient subgroups: Subgroup 1A (*n* = 69) and Subgroup 1B (*n* = 32) (Figure 4A). An isotonic MDS plot illustrates visually the distribution of these two sub-groups (Figure 4B), although there is a lower distinction between them compared to the distinction between Groups 1 and 2 (Figure 2B). Quantitatively, the internal clustering quality metrics (Figure 4B, connectivity = 47.35, Dunn index = 0.24) are both poorer than the clustering of Group 1 and 2 (Figure 2B), indicating that the distinction between the two subgroups is more subtle.

As predicted by the initial clustering analysis (Figures 2A,B), there was no significant difference in the magnitude of individual biological parameters between the two subgroups (Figure 4C and Figure S6). However, when patient characteristics in Subgroups 1A and 1B were analyzed, Subgroup 1A was characterized by significantly lower rates of flare, disease activity (SLEDAI-2k > 4 events as well as AMS being in the first quartile), SLE-related damage scores and treatment requirements (Table 2). Patients in this group also had significantly less renal, musculoskeletal, mucocutaneous, and immunological disease manifestations. This suggests that clustering based on the time-warped biological variables defined clinically meaningful subsets. Furthermore, when applying the previous time-dependent regression model in Subgroups 1A and 1B separately, the time-warped data had a significantly stronger ability to predict SLEDAI-2k in Subgroup 1A as compared to Group 1 overall (Figure 4D and Figures S7D,E).

**Table 2:** SLE patient demographic, clinical, and biological characteristics at baseline in subgroups 1A and 1B.

**Parameter**	**SLE Group 1 (*****n*** **=** **101)**		**Association OR (95% CI; *P*-value)**
	**SLE Group 1A (*n* = 69)**	**SLE Group 1B (*n* = 32)**	
**Sociodemographic characteristics**			
**Sex**			
Female	55 (80%)	28 (87.5%)	1.782 (0.576–6.738; 0.346)
Male	14 (20%)	4 (12.5%)	0.561 (0.148–1.736; 0.346)
**Ethnicity**			
Caucasian	39 (56.5%)	12 (38%)	0.462 (0.191–1.078; 0.078)
Asian	28 (40.5%)	18 (56%)	1.883 (0.81–4.456; 0.144)
Other/Missing	2 (3%)	2 (6%)	2.233 (0.258–19.337; 0.433)
**Disease characteristics**			
**Age at diagnosis (years)**			
<18 years	5 (7%)	6 (18.8%)	2.954 (0.822–11.076; 0.095)
≥18 to <45 years	49 (71%)	21 (65.6%)	0.779 (0.32–1.945; 0.585)
≥45 years	15 (22%)	5 (15.6%)	0.667 (0.2–1.928; 0.475)
**Duration of SLE (years)**			
<10 years	24 (35%)	12 (37.5%)	1.125 (0.464–2.672; 0.791)
≥10 years	45 (65%)	20 (62.5%)	0.889 (0.374–2.157; 0.791)
**Organ involvement (SLEDAI-2k)**			
Neurological	2 (3%)	4 (13%)	4.786 (0.882–35.965; 0.08)
Musculoskeletal	5 (7%)	12 (38%)	7.68 (2.53–26.66; 0.001)^*^
Renal	3 (4%)	7 (22%)	6.16 (1.581–30.349; 0.013)^*^
Mucocutaneous	10 (14%)	21 (66%)	11.264 (4.326–31.745; <0.001)^*^
Serositis	0 (0%)	2 (6%)	*Too few data points*
Immunological	13 (19%)	28 (88%)	30.154 (9.884–116.1; <0.001)^*^
Hematological	3 (4%)	4 (13%)	3.143 (0.653–16.835; 0.15)
**Adverse outcomes during observed period**			
SFI flare	45 (65%)	28 (88%)	3.733 (1.278–13.703; 0.026)^*^
SLICC-SDI ≥ 1	34 (49%)	23 (72%)	2.631 (1.093–6.758; 0.036)^*^
SLEDAI-2k > 4	44 (64%)	29 (91%)	5.492 (1.723–24.558; 0.009)^*^
AMS in 1st quartile (>4.96)	7 (10%)	19 (59%)	12.95 (4.716–39.461; <0.001)^*^
**Medications during observed period**			
Prednisolone	51 (74%)	32 (100%)	>1,000 (>1,000–∞; <0.001)^*^
Prednisolone >7.5 mg/day	39 (57%)	30 (94%)	11.54 (3.137–74.87; 0.001)^*^
Hydroxychloroquine	66 (96%)	31 (97%)	1.409 (0.173–29.111; 0.77)
Immunosuppressants	46 (67%)	32 (100%)	>1,000 (>1,000–∞; <0.001)^*^
Biologics	1 (1%)	6 (19%)	15.692 (2.518–304.031; 0.013)^*^

*Odds ratio (OR) calculated using penalized maximum likelihood logistic regression. OR were not calculated for rare events, where “Too few data points” is shown. AMS, time-adjusted mean SLEDAI-2k; SFI, SLE flare index*.

* *p-value < 0.05*.

## Discussion

SLE is a disease especially characterized by inter-patient heterogeneity and by time-dependent variation in disease activity, yet the variable of time is seldom analyzed formally in studies using biological parameters to identify patient subsets. If patients vary in the degree to which biological variables are concordant with clinical measures over time, analysis of pooled data without considering the variable of time (i.e., time agnostic analysis) risks failing to identify important patient subsets and/or associations. In our data, even a superficial assessment of the concordance between disease activity and a single biological marker in MIF (Figure 1), demonstrated that the degree of concordance between these variables over time ranges from high to zero between patients. This potentially explains weak associations between disease activity and biomarkers in many studies of pooled SLE patients. We therefore investigated whether a small set of biological parameters were associated with SLE disease activity differently when integrating time as a variable into a multi-dimensional analysis model, as a proof-of-concept for this approach. We found that while SLE patients could be stratified into subsets using routine magnitude-based clustering of biological parameters, the majority of patients were characterized by a multi-dimensional time-dependent association between biological parameters and disease activity, that was not evident when the effect of time was not considered. Together, these findings provide a proof-of-concept demonstration that statistically controlling for time-course variability in biological parameters may reveal clinically distinct patient groups and associations with disease activity in SLE, that would otherwise be hidden in the absence of these techniques.

Magnitude-based clustering of patient laboratory parameters, using data that was longitudinally collected but analyzed in a time-agnostic manner, identified two patient groups. One of these was small, accounting for <10% of the studied cohort. However, while patients from this group showed distinctly high inter-patient variability, they had significantly higher serum concentrations of MIF and the type I IFN-inducible chemokines CCL19, CCL2, and CXCL10. Because a type I IFN signature is reported in more than half of all SLE patients, and MIF is detectable in all, the presence of this signature alone cannot explain the delineation of patient Group 2. Rather, significantly increased serum levels of these cytokines suggest a distinctive immunological profile specific to this subpopulation of SLE patients, and indeed these biological parameters were strongly associated with overall disease activity in this subset. The high inter-patient variability defining this group suggests that more complete immune biomarker profiling in a larger cohort may reveal further clusters among SLE patients even leaving aside the element of time.

Longitudinal data present analytic challenges, but also opportunities to identify patient-stratifying biomarker patterns that are concealed when time is not considered. For example, two patients might have the same mean reading of a given variable, but very different dynamics over time. Approaches such as DTW (18) make it possible to analyse data that have a similar evolution but a different periodicity, a limitation of traditional longitudinal analysis in diseases such as SLE where disease fluctuations are highly variable between patients over time. In the majority of our cohort, biological parameters predicted disease activity only when integrating the time dimension, in a multi-dimensional analysis model. This approach also identified distinct subgroups, which were significantly different in rates of specific organ involvement, flare, disease activity and damage, indicating that this approach has the ability to reveal subsets that are clinically meaningful. It would be of value to evaluate whether discrepancies between studies regarding the clinical relevance of biomarkers [reviewed in (19)] may be linked to failure to incorporate the temporal dimension of the studied parameters.

There are caveats to the interpretation of this study. Firstly, although there were more than 100 well-characterized longitudinally followed SLE patients, this is a single center study and the approach needs to be independently validated. Secondly, only MIF and type I IFN-inducible chemokines were assessed; we predict that measurement of a larger cytokine expression panel would characterize additional SLE patient subsets, and indeed our findings provide proof-of-concept that including the dimension of time in such an approach adds additional insights. Further research is needed to evaluate which parameters are required to optimally characterize subsets of SLE patients, and also the optimal time sequence to be integrated for each biological parameter. Whether patient subsets identified by biological parameter time profiling are predictive of responses to treatments targeting the relevant pathways remains to be investigated, although differential responses to targeting the type I IFN pathway based on IFN biomarker status has been reported in Phase II clinical trials (20, 21), suggesting such opportunities may be imminent.

In conclusion, we confirmed that laboratory measurements of biological parameters including routine clinical pathology and novel chemokine biomarkers, analyzed in a multi-dimensional manner, can stratify SLE patients into distinct subsets. For the majority of patients, incorporating the factor of time revealed associations between biological parameters and disease activity that were not evident when only individual timepoints were examined. These findings indicate the potential for time-dependent analysis to enhance the identification of biologically distinct subsets of patients not evident using traditional longitudinal methods. This has implications for future SLE biomarker studies and stratification of patient subsets for receipt of targeted therapies. Potential applicability of these novel methods to other diseases characterized by time-dependent variability is suggested.

## Data Availability

The datasets generated for this study are available on request to the corresponding author.

## Ethics Statement

This study was carried out in accordance with the recommendations of the Human Research Ethics Committee, Monash Health (Monash Health HREC Reference 15526L) with written informed consent from all subjects. All subjects gave written informed consent in accordance with the Declaration of Helsinki. The protocol was approved by the Human Research Ethics Committee, Monash Health.

## Author Contributions

HN, KC, SB, EM, and FV wrote the main manuscript text. FP and SB performed initial clustering analyses. HN performed final statistical analyses and prepared the tables and figures. KC collected all data used in this study. All authors reviewed and approved the manuscript prior to submission.

### Conflict of Interest Statement

The authors declare that the research was conducted in the absence of any commercial or financial relationships that could be construed as a potential conflict of interest.
