# Supplementary material for: Novel Methods of Incorporating Time in Longitudinal Multivariate Analysis Reveals Hidden Associations With Disease Activity in Systemic Lupus Erythematosus
Source: Front Immunol. 2019 Jul 17;10:1649. doi: 10.3389/fimmu.2019.01649 (PMC6653068; doi:10.3389/fimmu.2019.01649)
Supplement: Supplementary file 1 [file Data_Sheet_1.docx]

**NOVEL METHODS OF INCORPORATING TIME IN LONGITUDINAL MULTIVARIATE ANALYSIS REVEALS HIDDEN ASSOCIATIONS WITH DISEASE ACTIVITY IN SYSTEMIC LUPUS ERYTHEMATOSUS**

Hieu T. Nim^1,2^^, Kathryn Connelly^2^^, Fabien B. Vincent^2^, François Petitjean^1^, Alberta Hoi^2^, Rachel Koelmeyer^2^, Sarah E. Boyd^2^, Eric F. Morand^2*^

^1^Data Science & AI, Faculty of Information Technology, Monash University, Clayton, Victoria, 3800, Australia

^2^Centre for Inflammatory Diseases, Monash University School of Clinical Sciences at Monash Health, Melbourne, Victoria, 3168, Australia

^^^ Equal contributions

Correspondence:

Professor Eric Morand

Telephone: +61 3 8572 2650. Fax: + 61 3 9594 6437

Email: [eric.morand@monash.edu](mailto:eric.morand@monash.edu)

**Supplementary Tables**

**Table S1.** SLE patient demographic, clinical and biological characteristics at baseline in subgroups 1 and 2.

| **Parameter** | **SLE Group 2 (n=110)** | | **Association of Group Label with Parameter**  OR (95% CI; P value) | |
| --- | --- | --- | --- | --- |
|  | **SLE Group 1**  (n = 101) | **SLE Group 2 2**  (n = 9) |  |  |
| ***Sociodemographic characteristics*** | | | |  |
| **Sex**  Female  Male | 83 (82%)  18 (18%) | 8 (89%)  1 (11%) | 1.735 (0.291 - 33.214; 0.614)  0.576 (0.03 - 3.435; 0.614) |  |
| **Ethnicity**  Caucasian  Asian  Other/Missing | 51 (50%)  46 (46%)  4 (4%) | 2 (22%)  7 (78%)  0 (0%) | 0.28 (0.04 - 1.225; 0.123)  (0.956 - 29.021; 0.083)  *Too few data points* |  |
| ***Disease characteristics*** | | | |  |
| **Age at diagnosis (years)**  <18 years  ≥18 - <45 years  ≥45 years | 11 (11%)  70 (69%)  20 (20%) | 2 (22%)  7 (78%)  0 (0%) | 2.338 (0.322 - 11.23; 0.325)  1.55 (0.351 - 10.802; 0.598)  <0.001 (0 – 0.001; <0.001) | * |
| **Time since diagnosis of SLE (years)**  <10 years  ≥10 years | 36 (36%)  65 (64%) | 4 (44%)  5 (56%) | 1.444 (0.339 - 5.792; 0.601) 0.692 (0.173 - 2.949; 0.601) |  |
| ***Disease characteristics continued*** | | | |  |
| **SLEDAI-2k organ domain**  Neurological  Vascular  Musculoskeletal  Renal  Mucocutaneous  Serosal  Immunological  Fever  Haematological | 6 (6%) 4 (4%)  17 (17%)  10 (10%)  31 (31%)  2 2%)  41 (41%)  1 (1%)  7 (7%) | 1 (11%) 0 (0%)  5 (56%)  3 (33%)  8 (89%)  0 (0%)  9 (100%)  0 (0%)  1 (11%) | 1.979 (0.099 - 13.765; 0.55) *Too few data points*  6.176 (1.491 - 27.313; 0.012)  4.55 (0.86 - 20.338; 0.053)  18.065 (3.125 - 342.551; 0.008)  *Too few data points*  >1000 (>1000 - ∞; <0.001)  *Too few data points*  1.679 (0.084 - 11.282; 0.647) | *  *  * |
| **Adverse outcomes during observed period**  SFI Flare  SLICC-SDI ≥ 1  SLEDAI-2k > 4  AMS in 1^st^ quartile (>4.96)  **Medications during observed period^^^**  Prednisolone  Prednisolone>7.5 mg/day  Hydroxychloroquine  Immunosuppressants  Biologics | 73 (72%)  57 (56%)  73 (72%)  26 (26%)  83 (82%)  69 (68%)  97 (96%)  78 (77%)  7 (7%) | 7 (78%)  7 (78%)  9 (100%)  2 (22%)  9 (100%)  7 (78%)  9 (100%)  7 (78%)  0 (0%) | 1.342 (0.302 - 9.377; 0.723)  2.702 (0.617 - 18.739; 0.229)  >1000 (>1000 - ∞; <0.001)  0.824 (0.118 - 3.672; 0.817)  >1000 (>1000 - ∞; <0.001)  1.623 (0.368 - 11.305; 0.559)  >1000 (>1000 - ∞; <0.001)  1.032 (0.23 - 7.247; 0.97)  <0.001 (0 – 0.001; <0.001) | *  *  * |

Odds ratio (OR) calculated using penalized maximum likelihood logistic regression. OR is not calculated for rare events, where “Too few data points” is shown.

^^^Restricted to medications taken by ≥10% of patients.

**Supplementary Figures**

**Figure S1**. A heat map based on the pairwise Euclidean distances of the patient pathology profiles (n=110). Dissimilarity values were normalised to [0-1], and plotted as a yellow-red heat map.


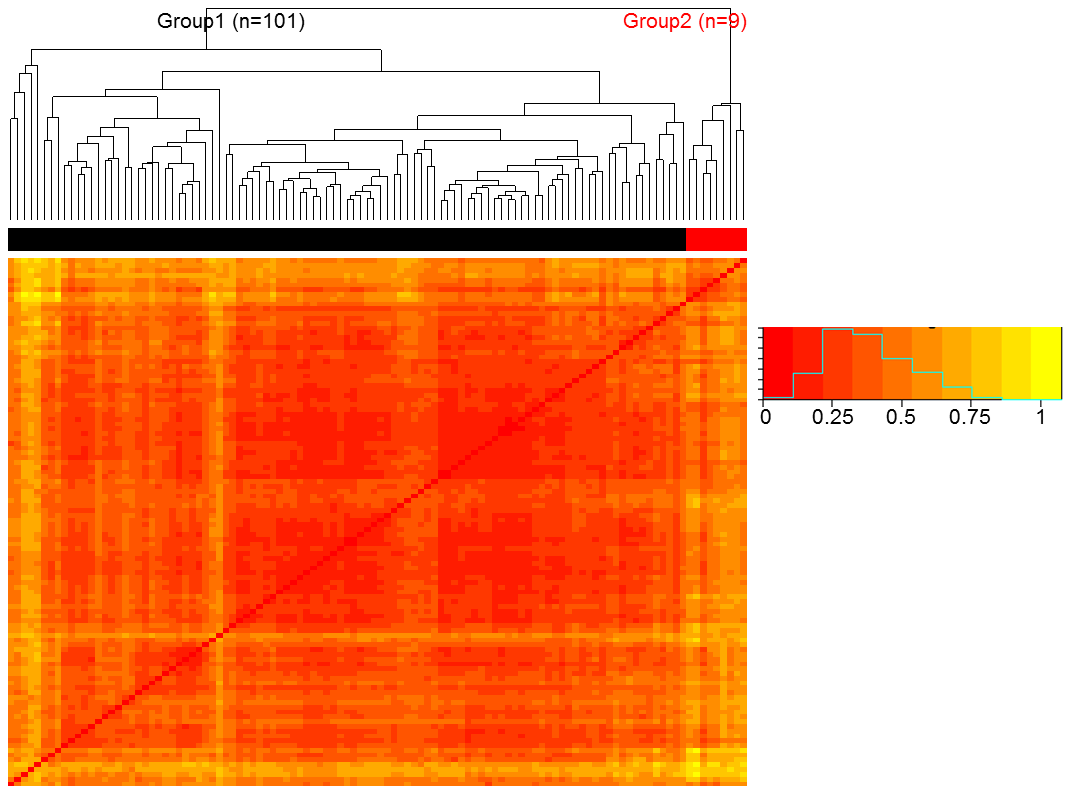


**Figure S2**. Boxplot comparison between the two patient subgroups (Group 1 and 2), based on 13 z-normalised blood and urinary parameters (excluding serum cytokines). Both t-test and Fisher’s exact test showed no statistical significance between Group 1 and Group 2 (p≥0.05).


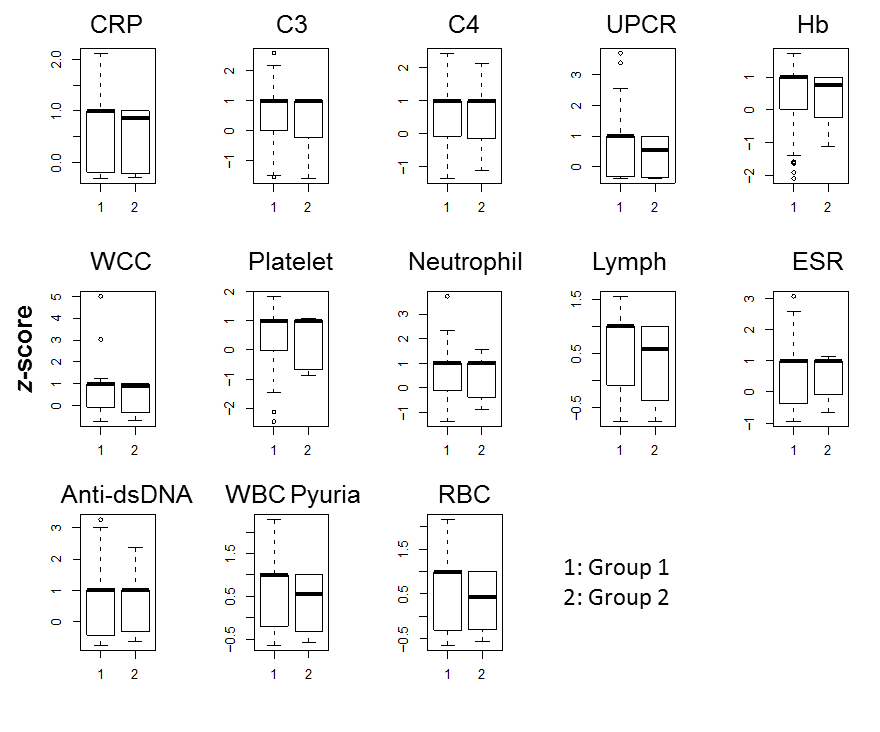


**Figure S3**. Results from LOPO multiple linear regression to predict the disease activity (SLEDAI-2K) of each patient visit based on the blood and urinary parameters, performed on (A) all patients versus (B) patients from Group 1. (C) Comparison of prediction error of Group 1 patients versus all patients without grouping information. n.s.: not statistically significant.


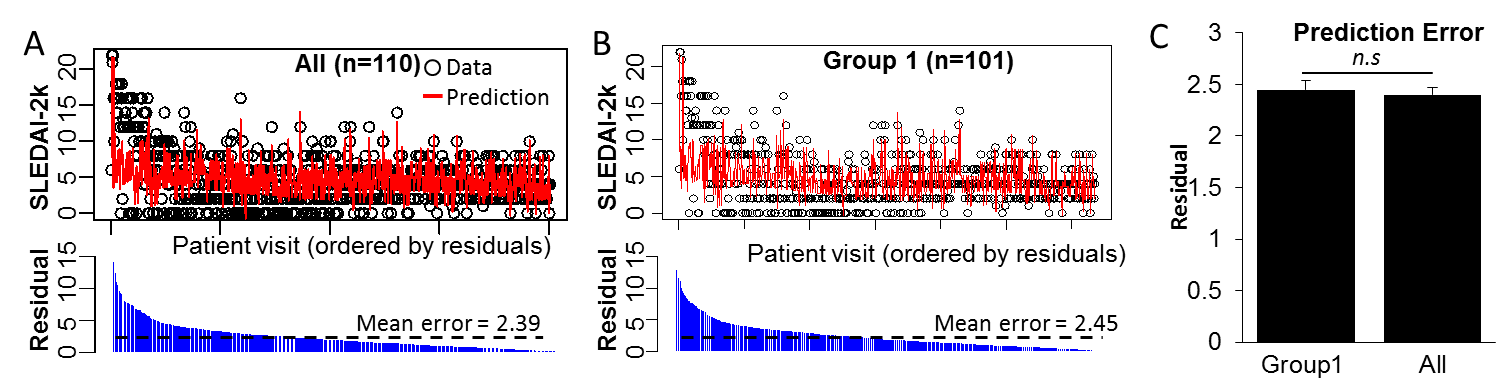


**Figure S4**. Boxplot comparison between the two patient subgroups (Group 1A and 1B), based on 13 z-normalised blood and urinary parameters (excluding serum cytokines). Both t-test and Fisher’s exact test showed no statistical significance between Group 1 and Group 2 (p≥0.05).


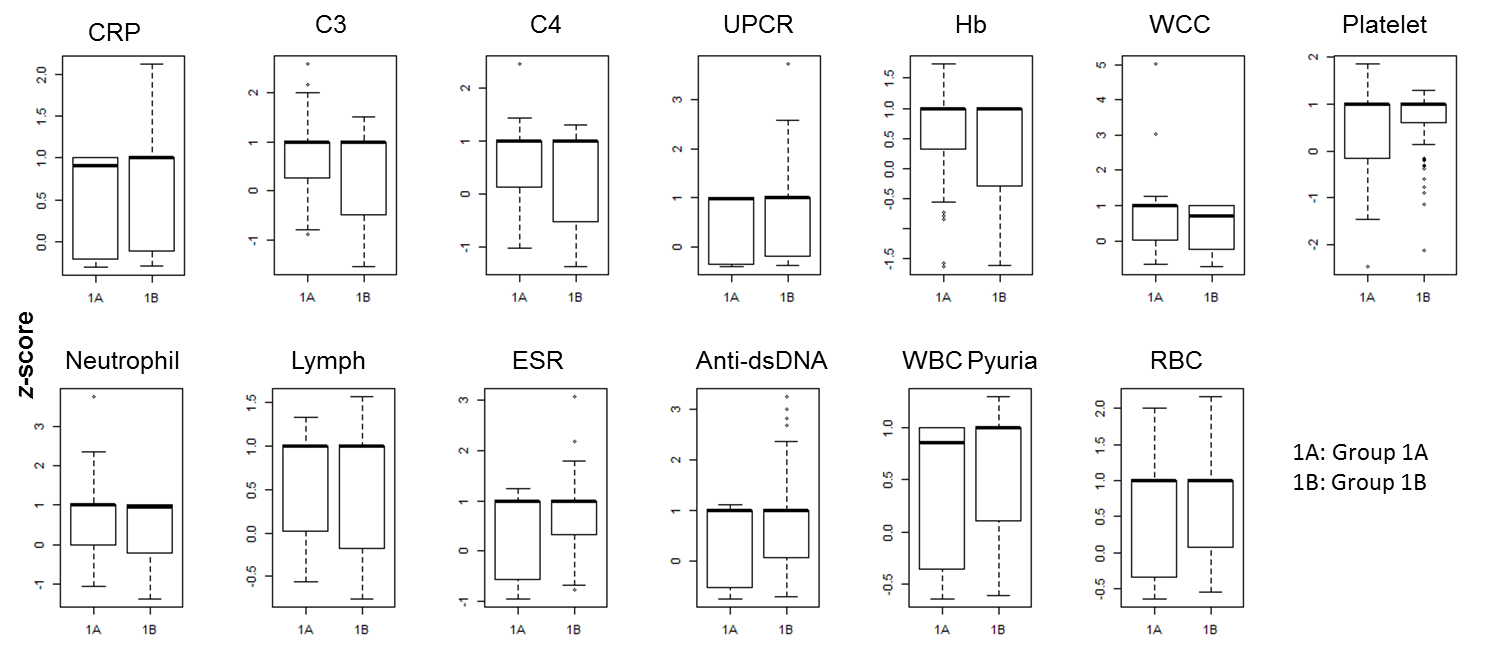


**Figure S5**. Ensemble clustering (using Ward, McQuitty, Centroid, and Median methods) as applied as the whole SLE patient cohort (n=110). All 4 clustering methods showed the highest likelihood of having 2 clusters.


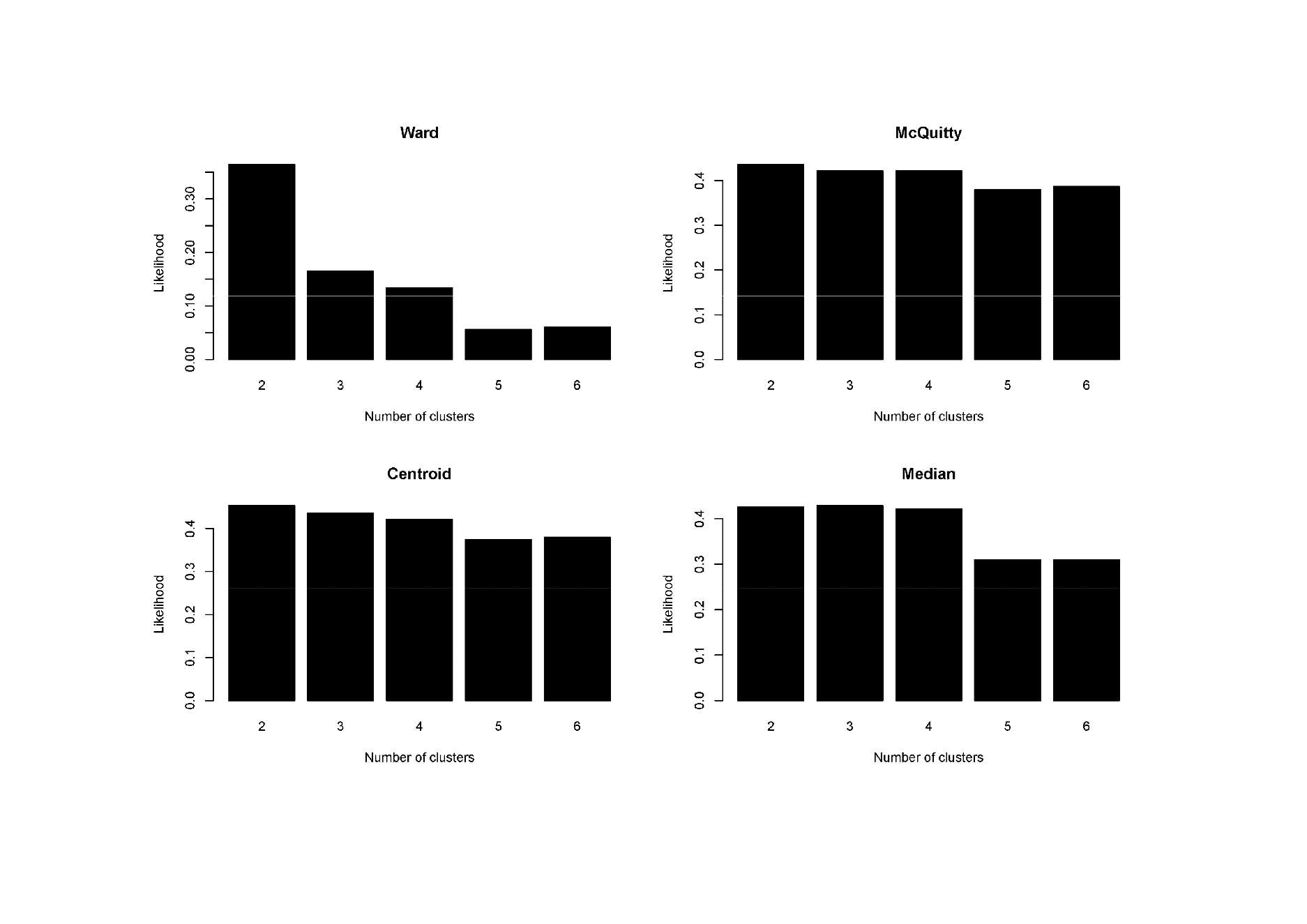


**Figure S6**. Ensemble clustering (using Ward, McQuitty, Centroid, and Median methods) as applied as Group 1 (n=101). Three out of four clustering methods showed the highest likelihood of having 2 sub-clusters within Group 1.


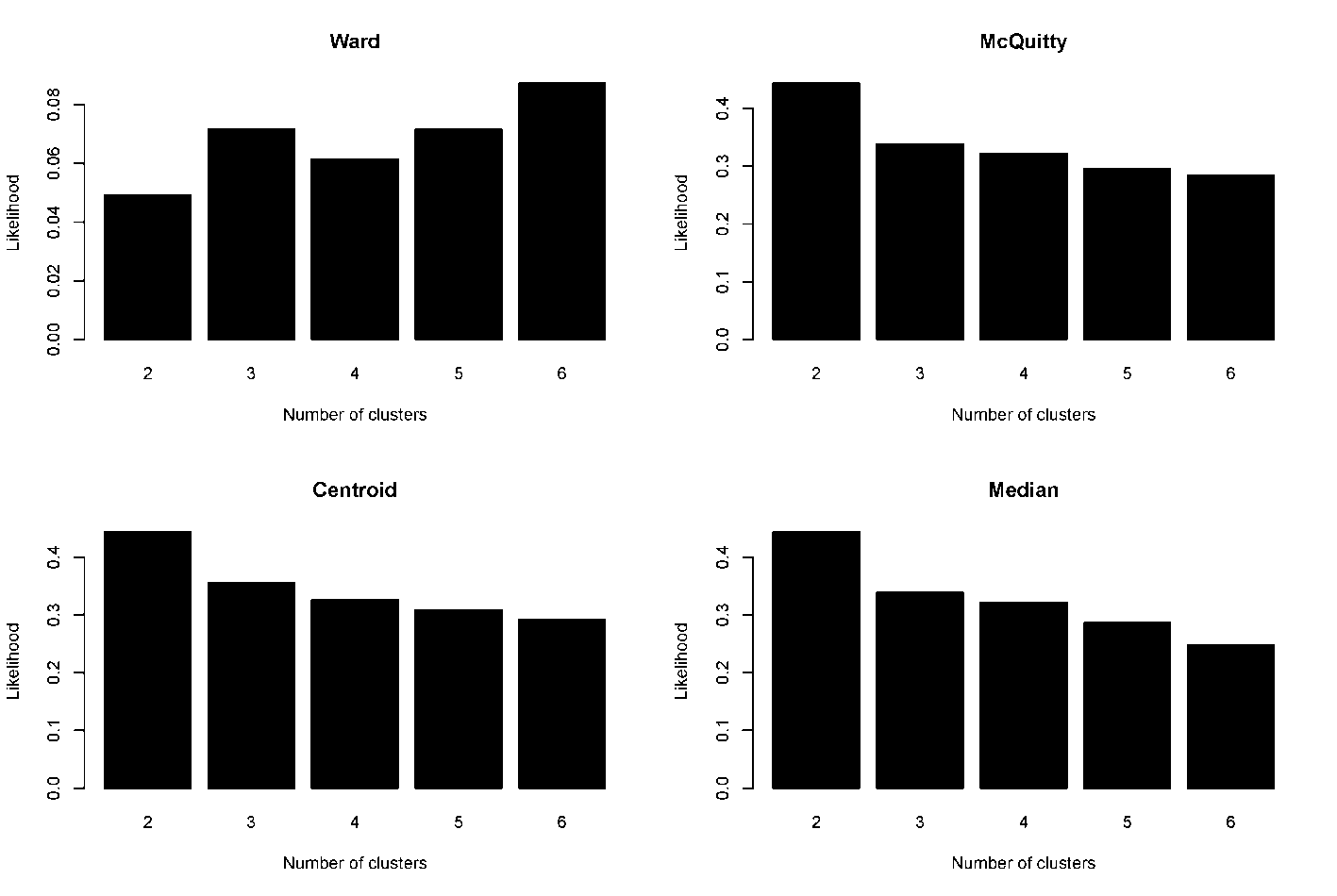


**Figure S7**. Results from boostrapping for multiple linear regression (80% training, 20% test data, 1000 iterations) to predict the disease activity (SLEDAI-2K) of each patient visit based on the blood and urinary parameters, performed on (A) All patients, (B) Group 1, (C) Group 2, (D) Subgroup 1A, and (E) Subgroup 1B.

**
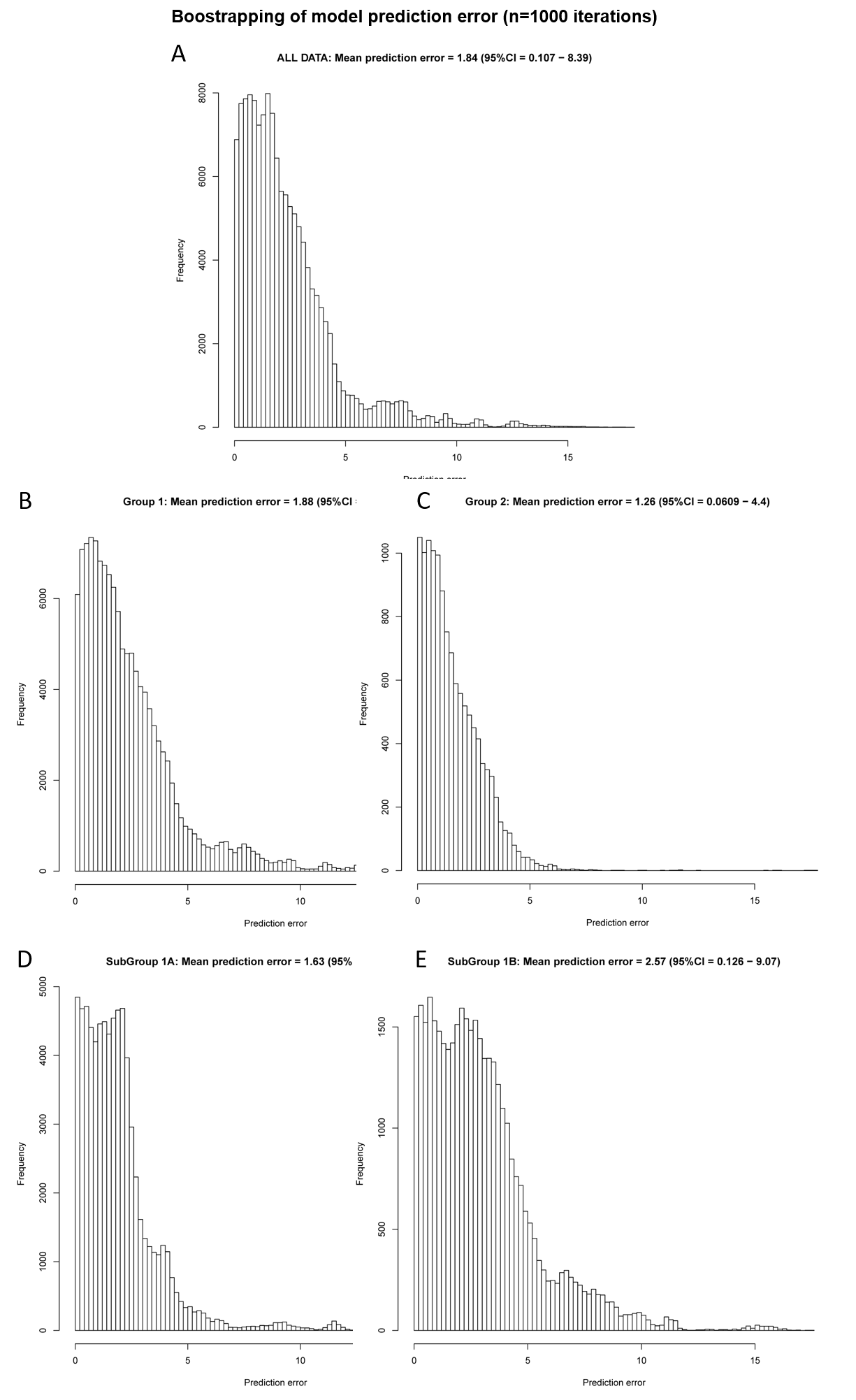
**
